# Cultural competency of GP trainees and GP trainers: a cross-sectional survey study

**DOI:** 10.1080/02813432.2023.2293927

**Published:** 2024-02-07

**Authors:** Siham Bouchareb, Amber A.W.A van der Heijden, Josine A.Y van Diesen, Maria van den Muijsenbergh, Sylvia Mennink, Henrica C.W de Vet, Annette H. Blankenstein, Petra J.M Elders

**Affiliations:** a Department of General Practice, Amsterdam UMC location Vrije Universiteit Amsterdam, Amsterdam, The Netherlands; b Amsterdam Public Health Research Institute, Amsterdam, The Netherlands; c Department of Medical and Clinical Psychology, Tilburg University, Tilburg, The Netherlands; d Department of Primary and Community Care, Radboud University Medical Centre, Nijmegen, The Netherlands; e Pharos, National Centre of Expertise on Health Disparities, Utrecht, The Netherlands; f Epidemiology and Data Science, Amsterdam UMC location Vrije Universiteit Amsterdam, Amsterdam, The Netherlands

**Keywords:** Cultural competence, general practice, migrants, GP trainees, GP trainers, survey

## Abstract

**Objective:**

To assess the cultural competence (CC) of GP trainees and GP trainers.

**Design and setting:** A cross-sectional survey study was conducted at the GP Training Institute of Amsterdam UMC.

**Subjects:**

We included 92 GP trainees and 186 GP trainers.

**Main outcome measures:**

We measured the three domains of cultural competency: 1) knowledge, 2) culturally competent attitudes and 3) culturally competent skills. Regression models were used to identify factors associated with levels of CC. Participants rated their self-perceived CC at the beginning and end of the survey, and the correlation between self-perceived and measured CC was assessed.

**Results:**

Approximately 94% of the GP trainees and 81% of the GP trainers scored low on knowledge; 45% and 42%, respectively, scored low on culturally competent attitudes. The level of culturally competent skills was moderate (54.3%) or low (48.4%) for most GP trainees and GP trainers. The year of residency and the GP training institute were significantly associated with one or more (sub-)domains of CC in GP trainees. Having >10% migrant patients and experience as a GP trainer were positively associated with one or more (sub-) domains of cultural competence in GP trainers. The correlation between measured and self-perceived CC was positive overall but very weak (Spearman correlation coefficient ranging from −0.1–0.3).

**Conclusion:**

The level of cultural competence was low in both groups, especially in the knowledge scores. Cultural competence increased with experience and exposure to an ethnically diverse patient population. Our study highlights the need for cultural competence training in the GP training curricula.

## KEY POINTS

General practitioner (GP) trainees find cross-cultural consultations stressful due to a self-perceived lack of cultural competence (CC). The level of CC in general practice is as yet unknown.

- On average, the level of CC was low for the majority of GP trainees and GP trainers, especially for the scores on knowledge.
- CC increased with experience and exposure to an ethnically diverse patient population.
- GP trainees and trainers perceived a lack of covered education on various topics related to the care of migrants.
- Our study highlights the need for cultural competence training in the GP training curricula.

## Introduction

Health care professionals (HCPs) provide care to an increasingly ethnically and culturally diverse patient population [1]. In the Netherlands, about 25% of the population has a migrant background, with higher proportions in major cities [2], largely represented by migrants from Turkey, Morocco, Surinam and refugees from the Middle East and Africa [2,3]. Migrant and ethnic minority populations have poorer health outcomes than native Dutch people of the same socioeconomic status [4–6]. Mutual understanding between general practitioners (GPs) and patients and adherence to treatment are more often poor in cross-cultural consultations [7,8]. GPs also tend to involve migrant patients less in decision-making and are less likely to check patient understanding in cross-cultural consultations [9]. A language barrier between HCPs and their patients can lead to decreased adequacy and quality of care and lower patient satisfaction [10–12].

These barriers to migrant care can be reduced through culturally competent care [13], defined as person-centred care with a range of specific knowledge, attitudes and skills necessary to provide good-quality care to an ethnically and culturally diverse patient population [14,15]. In clinical practice, this means that HCPs have knowledge of the relationship between disease and ethnic or socio-cultural factors, such as ethnic differences in pharmacokinetics. They also need person-centred attitudes (e.g. empathy, respect and curiosity) and skills to communicate effectively with, for example, patients with low health literacy and to overcome language barriers [14,15]. These factors can be improved through training in cultural competence [9,16,17]. However, a review [9] of studies from Australia, Canada, the United Kingdom, Sweden, Norway and the Netherlands reported that training in CC is underdeveloped in general practice.

GP trainees often find cross-cultural consultations more stressful due to their self-perceived lack of CC and generally want more training in culturally competent care [9]. In order to develop an educational programme for the GP training curricula that addresses the gaps in CC, it is important to gain insight into the level of CC of GP trainees and trainers, which is currently lacking. Therefore, we aimed to assess 1) the level of knowledge, attitudes and skills (i.e. CC) of GP trainees and GP trainers in the Netherlands regarding the care of migrants in general practice; 2) the relationship between personal factors and the assessed level of CC; 3) the relationship between assessed and self-perceived CC; and 4) the extent to which various topics related to the care of migrants were perceived as being covered.

## Methods

### Study design

We conducted a cross-sectional survey study among GP trainees and trainers in the general practice residency training programme at the Amsterdam University Medical Centres (AMC and VUmc sites) from August to September 2020.

### Development of the questionnaire

The theoretical basis for the development of the questionnaire was the conceptual framework for cultural competence developed by Seeleman et al. [15]. We followed their three domains of cultural competence: 1) knowledge: *knowledge* of epidemiology and the differential impact of treatment in different ethnic groups; 2) attitudes: *awareness* of how culture and migration shape individual behaviour and thinking; awareness of the social context of ethnic minority groups; and 3) skills: *the ability* to convey information in a way that the patient can understand, to use external help (e.g. interpreters) when needed, and to adapt flexibly and creatively to new situations. The framework was previously used by Seeleman et al. to develop a questionnaire on CC among medical students, youth health care physician residents and their supervisors [18].

An adapted version of Seeleman’s questionnaire [18] was recently used to assess CC among midwives in three different European countries [19]. In order to identify relevant topics for the development of our questionnaire suitable for GP trainees and GP trainers, we conducted a literature review on facilitators and barriers in the care of people with a migration background in the Netherlands. In addition, two books [20,21] on the care of migrants and socially vulnerable people in the Netherlands, based on scientific literature and best practices, were reviewed. One of these books is used in the Dutch GP residency programme, and the other [21] was found in the referenced literature.

The questionnaire was developed by the research team, which included a GP trainee with a migration background (SB), experienced GPs (AB and PE), two GPs who worked as teachers at the general practice residency training institute (AB and SM), one of whom had a special focus on diversity (SM), a GP and an expert in health disparities and person-centred integrated primary care (MvdM) and an expert in clinimetrics (HdV). The factors identified from the literature were discussed with three members of the research team, AB, PE and SB, and then used to create a first draft of the questionnaire. We operationalised the domains of cultural competence described above from the original framework into the questionnaire as follows (see Appendix 1):

1. Knowledge: general knowledge about ethnic minority care, such as pharmacotherapy, diabetes and Ramadan; care for undocumented patients and people with low (health) literacy; and knowledge about specific diagnoses that are particularly prevalent in certain ethnic groups.
2. Attitudes: culturally competent consultation attitudes, including awareness of the role of social context and patient-specific factors, such as health literacy, in relation to, for example, non-adherence; awareness of how culture shapes individual behaviour and thinking; and curiosity to understand or learn more about patients’ perspectives. We chose not to operationalise awareness of one’s own biases and tendency to stereotype because we believe that a survey is not appropriate to measure this sub-domain as it is likely to result in socially desirable responses.
3. Skills: culturally competent skills, which include the ability to adapt communication to patients’ (health) literacy, to assess when external help with communication is needed (e.g. an interpreter) and how to work with an interpreter in the consultation, to compromise with patients or their families when necessary, and to adapt flexibly and creatively to new situations.

All items on the questionnaire were thoroughly discussed by the researchers (AB, PE and SB) until a consensus was reached on their relevance to general practice. Based on the literature and clinical experiences of the researchers, we determined appropriate (correct) and inappropriate (incorrect) normative response options (answers) for items on culturally competent consultation attitudes and culturally competent skills. The expert panel of our research team (SM, MvdM and HdV) provided feedback on the first draft of the questionnaire. After consensus was reached on the first draft of the questionnaire, we pilot-tested the survey with three other GPs and a GP trainee in one-to-one online (Zoom) sessions, in which we used the so-called “think-aloud method” [22] to assess the comprehensibility, comprehensiveness and relevance of the questionnaire. In this session, participants were asked to complete the questionnaire aloud while ­sharing their thoughts about the questions.

The final questionnaire was adapted after pilot testing and consisted of 48 items divided into four main sections. The English translation of the questionnaire and a summary of the interpretation of the answers can be found in Appendix 2.

1. General questions: nine items on respondent characteristics such as ethnicity, work experience and affinity for caring for patients with a migration background.
2. Medical cases: a total of eight multiple-choice questions on culturally competent consultation attitudes or culturally competent skills.
3. Knowledge questions: the first part comprised nine items on knowledge about caring for people with a migration background (“true”, “false” or “I do not know”). The second part concerned specific diagnoses and consisted of six short cases of medical conditions that are highly prevalent in certain ethnic groups. Respondents were asked to make a differential diagnosis (open questions).
4. Education: 11 items on the extent to which respondents felt they had received educational training on the specific topics mentioned (1 = not covered, 2 = inadequately covered, 3 = adequately covered), and whether they want more education about these topics (yes/no). Educational needs (not included in the analysis described in this paper).

In addition, participants were asked at the beginning and end of the questionnaire to what extent they felt culturally competent in providing care to patients with a migration background (i.e. self-perceived CC) on a scale of 1–10. For the purposes of this study, migrants were defined as people with a non-Western migration background. These are people originally from Turkey, Africa, Latin America and Asia, including asylum seekers, refugees and undocumented people.

### Participants and procedure

In the Netherlands, general practice residency consists of a three-year training programme. In the first and third years of training, GP trainees work in general practice under the supervision of a GP trainer. In the second year, trainees have internships in a variety of settings. GP trainers are experienced GPs who receive regular training in supervising GP trainees in general practice. For this study, we invited 627 GP trainers from the two GP training programmes at Amsterdam UMC (VUmc and AMC sites) and 343 GP trainees who were actively working in general practice to participate in an anonymous questionnaire. In order to ensure the anonymity of our participants, an email with a link to the questionnaire was sent by the secretariat of the general practice training institutes. As GP trainees in their second (middle) year of training do not work in general practice, we decided to exclude them and only invite trainees in their first or last year of training. Reminder emails were sent after two and four weeks after the first email. Respondents were not compensated for their participation in the study.

### Educational programmes at the GP training at Amsterdam UMC

The GP trainees at Amsterdam UMC receive training one day a week at the training institute under the supervision of a GP teacher and a behavioural science teacher [23,24]. This training day mainly consists of a reflection session on their experiences in general practice, education in the group on topics that they can freely choose according to their learning needs, and so-called thematic education (e.g. care for migrants). Thematic education is usually given by a guest teacher with expertise of the specific topic. Additionally, GP trainees can choose from a list of topics which specific thematic educational session they would like to attend. Therefore, not all trainees receive education on the same topics. In recent years, both GP training institutes at Amsterdam UMC have started to integrate the topics of diversity and migrant care into their curricula. For example, the VUmc GP training institute has a website with articles, podcasts and other relevant literature on caring for migrants embedded in the themes of “diversity”, “chronic care” and “doctor-patient communication” [25].

The website contains materials that trainees can use to learn about topics such as intercultural palliative care (video “I have a doctor in Morocco”), diabetes and Ramadan, and the care of undocumented patients. GP trainees have the opportunity to attend different thematic trainings relevant to the care of migrants, such as low health literacy and diversity in emergencies. The AMC training institute offers the following educational programmes and materials for GP trainees: ethnic differences in skin diseases, diabetes and Ramadan, treatment of hypertension in patients of sub-Saharan African descent, palliative care and migrant-specific aspects of symptom management, low health literacy, use of an interpreter and medically unexplained physical symptoms (MUPS) in migrants.

### Scores and interpretation

The maximum scores for knowledge, culturally competent consultation attitudes and skills were 15, 3 and 5 points, respectively. The mean scores of knowledge, culturally competent consultation attitudes and skills, and their sub-domains were expressed as a proportion of the maximum score per domain and per sub-domain. The mean scores were categorised as good (>80% of the maximum score), moderate (60–80% of the maximum score) and low (<60% of the maximum score).

### Analysis

Descriptive statistics were used to summarise respondent characteristics and questionnaire responses. Continuous variables were presented as mean: ±SD or 95% confidence intervals (CI) for normally distributed data or as median [IQR] for skewed data, and categorical variables were presented as numbers (proportions). The difference between self-perceived CC at the beginning (CC1) and at the end (CC2) of the questionnaire was tested with a paired *t*-test. We used Spearman’s correlation coefficient (95% CI) for the correlation between self-perceived CC scores and the scores of each domain of CC. Linear regression analysis with a backward selection procedure was performed to determine the most important factors for the levels of the different CC domains. Factors included in the models were respondents’ demographic data, work-related factors and affinity for caring for migrants. Ordinal variables that existed in five categories were merged into three categories to increase the statistical power of the models.

The variables CC1, proportion of migrants, and ethnicity were merged into dichotomous variables on the basis of distribution and logical reclassification. For example, the Dutch and European origins of the variable “ethnicity” were merged into one category. Continuous variables that met the assumptions of linearity were included in the models as continuous variables; otherwise, they were dummy variables. Collinearity between the independent variables was tested using Spearman’s correlation coefficient and collinearity statistics (i.e. tolerance and VIF). Associations were considered statistically significant at a *p*-value of ≤ 0.05, and we chose a threshold of ≤ 0.10 for the regression analysis. Data analysis was stratified for GP trainees and GP trainers and performed using SPSS version 26.

### Medical, ethical and legal aspects

The Medical Ethics Review Committee of the VU University Medical Centre (Amsterdam UMC) confirmed that the study did not fall within the scope of the Medical Research Act on Human Subjects; therefore, committee approval was not required (2020.404). All participants in the think-aloud sessions gave their consent for the session to be audio-recorded. At the end of the questionnaire, respondents were asked if they would be willing to contribute to the development of an educational programme based on the results of the survey study. Those who wished to provide input were asked for their email addresses, which were immediately separated from the data to ensure the anonymity of the participants.

## Results

### Response rate and characteristics of participants

The response rate was 26.8% for GP trainees and 29.7% for GP trainers. Table 1 shows that most GP trainees (60.9%) were in their first year of residency. The average work experience of GP trainers was 20 years as a GP and eight years as a GP trainer. About 50% of GP trainees and GP trainers reported seeing less than 10% of migrant patients in their general practice; 80% of both groups were of Dutch descent. An affinity for the care of migrant patients was mentioned by 27% and 32% of GP trainees and GP trainers, respectively. 86% of the GP trainees and 64% of the GP trainers agreed or strongly agreed that they experienced barriers in caring for patients with a migration background due to language and/or cultural differences.

**Table 1.** Participant characteristics and information about caring for people with a migration background.

	GP trainees *N* = 92	GP trainers *N* = 186
Female	72 (78.3%)	104 (55.9%)
GP training institute		
- VUmc	46 (50.0%)	92 (49.5%)
- AMC	46 (50.0%)	94 (50.5%)
Year of residency		N.A.
- First year	56 (60.9%)	
- Third [last] year	36 (39.1%)	
Years of experience as a junior doctor before the start of GP training	2.5 [2.0–4.0]	N.A.
Years of experience as a GP trainer	N.A.	8.0 [4.0-14.0]
Years of experience as a GP	N.A.	20.0 (±7.5)
Ethnicity of participants^a^		
- Dutch origin	71 (77.2%)	155 (83.8%)
- European origin	3 (3.3%)	11 (5.9%)
- Origin outside Europe	18 (19.6%)	19 (10.3%)
Migrant patients in general practice		
-≤10%	46 [50.0%]	84 (45.2%)
−11–25%:	18 [19.6%]	46 (24.7%)
−26–50%	21 (22.8%)	41 (22.0%)
- >50%	7 (7.6%)	15 (8.1%)
Countries of origin of migrant patients mentioned in the top 3^b^		
- Turkey	59 (68.6%)	125 (69.4%)
- Morocco	47 (54.7%)	130 (72.2%)
- Syria	30 (34.9%)	52 (28.9%)
Affinity with care of migrant patients		
- Special affinity	6 (6.5%)	11 (5.9%)
- Affinity	25 (27.2%)	60 (32.3%)
- Neutral	45 (48.9%)	95 (51.1%)
- Little affinity	13 (14.1%)	20 (10.8%)
- No affinity	3 (3.3%)	0 (0.0%)
Perceived barriers due to language and/ or cultural differences		
- Strongly disagree	1 (1.1%)	7 (3.8%)
- Disagree	3 (3.3%)	29 (15.6%)
- Neutral	9 (9.8%)	31 (16.7%)
- Agree	71 (77.2%)	106 (57.0%)
- Strongly agree	8 (8.7%)	13 (7.0%)

Mean (±SD) for normally distributed continuous variables.

Median [IQR] for skewed distributed continuous variables.

N (%) for categorical variables.

^a^ 1 (0.4%) missing in the GP supervisors group.

^b^ 35 (11.6%) missing of the total number of participants (*N* = 278).

### Assessment of cultural competence

Table 2 shows the scores for the questions relating to the three domains of CC and their subsections, classified as low, moderate or high. In our scoring system, the vast majority of GP trainees (93.5%) and GP trainers (80.6%) had a low level of knowledge on average. Almost half of GP trainees (44.6%) and GP trainers (42.5%) scored low on culturally competent consultation attitudes. The scores for culturally competent skills were moderate (54.3%) and low (48.4%) for most GP trainees and GP trainers, respectively. The mean (95% CI) score of self-perceived CC at the beginning of the questionnaire was 6.2 (5.9–6.5) and 6.3 (6.1–6.5) among GP trainees and GP trainers, respectively.

**Table 2.** Scores on knowledge, culturally competent consultation attitudes and skills and their sub-sections for GP trainees and GP trainers.

	Interpretation of scores^a^ GP trainees			Interpretation of scores^a^ GP trainers		
	Low level N (%)	Moderate level N (%)	High level N (%)	Low level N (%)	Moderate level N (%)	High level N (%)
Knowledge	86 (93.5%)	6 (6.5%)	0 (0.0%)	150 (80.6%)	36 (19.4%)	0 (0.0%)
- General knowledge	85 (92.4%)	7 (7.6%)	0 (0.0%)	172 (92.5%)	13 (7.0%)	1 (0.5%)
- Specific diagnosis	57 (62.0%)	32 (34.8%)	3 (3.3%)	99 (53.2%)	65 (34.9%)	22 (11.8%)
Culturally competent consultation attitudes	41 (44.6%)	38 (41.3%)	13 (14.1%)	79 (42.5%)	77 (41.4%)	30 (16.1%)
- Awareness of social context and patients’ specific factors	51 (55.4%)	25 (27.2%)	16 (17.4%)	89 (47.8%)	48 (25.8%)	49 (26.3%)
- Awareness of how culture shapes an individual’s behaviour and thinking, and curiosity about understanding or learning more about patients’ perspectives	22 (23.9%)	33 (35.9%)	37 (40.2%)	57 (30.6%)	61 (32.8%)	68 (36.6%)
Culturally competent skills [range score 0-5]	28 (30.4%)	50 (54.3%)	14 (15.2%)	90 (48.4%)	82 (44.1%)	14 (7.5%)
-The ability to adapt communication to patients’ [health] literacy, to assess when external help with communication is needed and how to work with an interpreter in consultation	32 (34.8%)	41 (44.6%)	19 (20.7%)	113 (60.8%)	53 (28.5%)	20 (10.8%)
-The ability to compromise with patients or their family as needed, to adapt flexibly and creatively to new situations	42 (45.7%)	30 (32.6%)	20 (21.7%)	89 (47.8%)	52 (28.0%)	45 (24.2%)

^a^ interpretation scores: <60% of maximum score = low; 60–80% of maximum score = moderate; >80% of maximum score = high score.

At the end of the questionnaire, GP trainees and GP trainers rated their self-perceived CC slightly but sta­tistically significantly lower at 5.8 (5.5–6.0) and 6.0 (5.8–6.2), respectively. The correlations between self-perceived CC (CC1 and CC2) and assessed CC were all positive and very weak. The strongest correlations were found between general knowledge and self-perceived CC2 among GP trainees (rs = 0.3; 0.1–0.5) and GP trainers (rs = 0.3; 0.2–0.4). The weakest correlations were found between the ability to compromise with patients or their families and CC1 (rs = 0.1; −0.2–0.3) and CC2 (rs = −0.1; −0.3–0.2) in GP trainees and between the ability to adapt communication to patients’ literacy and CC2 (rs = 0.1; −0.1–0.2) in GP trainers.

### Relationship between several variables and assessed cultural competence

Among GP trainees, the last year of residency and AMC GP training institute were significantly associated with higher levels of knowledge. However, this training institute was also associated with lower levels of culturally competent skills among GP trainees (Table 3). Among GP trainers, a proportion of more than 10% of patients with a migrant background was significantly associated with a higher level of knowledge and a higher level of culturally competent consultation attitudes. Coming from a non-European country was also positively associated with the level of knowledge among GP trainers. Among GP trainers, years of work experience as a GP trainer (3^rd^ quartile) was significantly associated with higher levels of culturally competent skills. The last year of residency and a non-European ethnicity were associated with higher levels of culturally competent skills among GP trainees.

**Table 3.** The relationship between several factors and the assessed cultural competence.

	Knowledge		Culturally competent consultation attitudes		Culturally competent skills	
	GP trainees	GP trainers	GP trainees	GP trainers	GP trainees	GP trainers
	Beta (95%CI)	Beta (95%CI)	Beta (95%CI)	Beta (95%CI)	Beta (95%CI)	Beta (95%CI)
Sex	–	–	–		–	–
0 Female						
1 Male						
GP training institute		–	–			–
0 VUmc	–				–	
1 AMC	0.8 (-0.1 − 1.6)*				−0.3 (-0.6 − 0.0)*	
Year of residency		–	–			–
0 First year of residency	–				–	
1 Last year of residency	1.5 (0.6 − 2.4)*				0.3 (-0.0– 0.6)*	
Years of experience as a junior doctor before the start of GP training^a^	–	N.A.	–	N.A.	–	N.A.
Years of experience as a GP trainer	N.A.	–	N.A.	-	N.A.	
0 Quartile1						–
1 Quartile2						0.2 (-0.1 − 0.6)
2 Quartile3						0.4 (0.0 − 0.7)*
3 Quartile4						−0.0 (-0.3 − 0.3)
Years of experience as a GP	N.A.	–	N.A.	–	N.A.	–
0 Quartile1						
1 Quartile2						
2 Quartile3						
3 Quartile4						
Percentage of migrant patients in general practice	–		–		–	–
0 ≤ 10%		–		–		
1 > 10%		1.6 (0.9 − 2.3)*		0.3 (0.1 − 0.4)*		
Ethnicity of participants	–	–	–	–	–	–
0 European origin						
1 Origin outside Europe		1.1 (-0.1–2.2)*			0.4 (0.0–0.8)*	
Affinity with care of migrant patients^b^	–	–	–	–	–	–
0 No affinity						
1 Neutral						
2 Affinity						
Self-perceived CC1	–	–	–		–	–
0 Score 1-5				–		
1 Score ≥6				0.2 (0.0 − 0.4)*		

0= the reference group.

* Significance at *p* ≤ 0.10.

^a^ Continuous variable.

^b^ The categorical variable perceived barriers in care of migrants was excluded from the analysis due to collinearity with affinity with care for migrants in both groups.

### Perceived education

**Figure 1.**
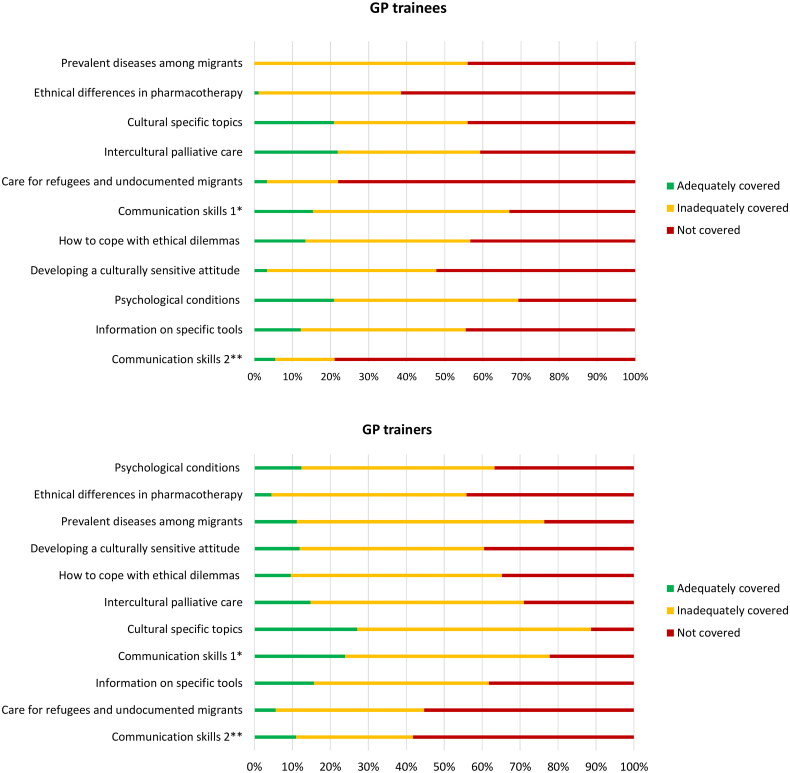
Perceived education of GP trainees and GP trainers in relation to migrant health care, according to the questionnaire. ^a^Communication skills to overcome language barriers and functional illiteracy, such as clarifying the request for help, exploring concerns and emotions, and giving explanations tailored to the patient’s level of literacy. ^b^Communication skills for holding consultations with an interpreter (e.g. maintaining control).

Figure 1 provides an overview of the extent to which education was provided on various topics relevant to the care of migrants. The vast majority of GP trainees and trainers reported that the topics were either not covered or were covered inadequately during the GP residency programme, a refresher day or during continuing education. Additional sub-analyses showed that VUmc GP trainees reported more frequently that most topics were not covered compared with AMC GP trainees. For example, almost 75% of VUmc GP trainees compared with 50% of the GP trainees at AMC reported that the topic “ethnical differences in pharmacotherapy” was not covered. There were no major differences between VUMC and AMC, the GP trainers, in terms of the amount of education provided (see Appendix 3).

## Discussion

This survey investigated CC among GP trainees and GP trainers. Participants’ levels of CC were low on average, particularly scores for “general knowledge of ethnic minority care provision” and “specific diagnosis”. Our findings are consistent with a survey study [18] of medical students, youth health care physician residents and their supervisors, which also found generally low scores in the knowledge domains. These low scores in CC may be explained by the fact that the training institutes of Amsterdam UMC have only recently started to develop and structurally embed educational programmes related to the care of migrants in the GP training curricula. In addition, many of our respondents in both groups indicated a perceived lack of training in migrant care. Training in CC is also lacking in general practice internationally [9]. A qualitative study [26] of GP trainees in Ireland also found that the majority felt that training in the care of migrant patients was inadequate and that the main need was for factual knowledge about caring for an ethnically and culturally diverse population. Studies have shown that CC training could improve CC in HCPs [19,27,28]. For example, a recent study of midwives reported significant improvements in knowledge, skills and self-perceived CC after training in culturally sensitive maternity care [19].

Our participants generally scored slightly higher on culturally competent consultation attitudes and skills, particularly in the sub-domains of awareness of culturally dependent behaviour and interest in patient perspectives. The emphasis on patient-centred communication in GP training may explain the higher scores. The AMC training institute was positively associated with knowledge and VUmc with culturally competent consultation skills. These results could be explained by the possible differences in the curricula of the two GP training institutes. The GP training institute of the VUmc seems to have educational programmes with an emphasis on diversity in doctor-patient communication, such as low health literacy and intercultural palliative care. On the other hand, the GP training curriculum of the AMC appears to have a stronger focus on knowledge compared to that of the VUmc, with educational topics such as ethnic differences in skin diseases, treatment of hypertension in patients of sub-Saharan African descent, as well as palliative care and migrant-specific aspects in symptom management. The sub-analyses showed that the vast majority of trainees at both GP institutes reported that the various topics related to the care of migrants were either not covered or were covered inadequately. However, it appears that GP trainees at the AMC were more likely than those at the VUmc to perceive that various topics were inadequately or to a lesser extent adequately covered. For the development of educational programmes on CC, it will be interesting to explore these dissimilarities more thoroughly, for example, through focus groups, given the possible differences in the curricula of these institutes.

In addition, the positive association between the last year of residency and the level of knowledge and culturally competent skills of GP trainees is probably a reflection of their increased experience. In GP trainers, having a patient population with more than 10% migrants and work experience as a GP was also found to be positively associated with the level of CC. These findings are coherent with studies that reported on the positive relationship between exposure to a culturally diverse population, work experience, and the development of CC [9,29]. Moreover, almost half of the GP trainers indicated having 10% or fewer patients with a migration background in general practice, which might explain their low level of CC. The associations between self-perceived CC and the assessed domains of CC were all very weak. Similar results were found in previous studies [18,30], which highlighted the importance of assessing CC beyond participants’self-perception [31,32]. A systematic review [30] demonstrated little, no, or an inverse association between self-perceived and observed competence among physicians in most studies. In addition, we found that self-perceived CC1 (at the beginning of the questionnaire) was significantly lower compared to self-perceived CC2 (at the end of the questionnaire), which might indicate that in the process of completing the questionnaire, participants became aware of their incompetence. It has been shown that HCPs are often unaware of their incompetence regarding care for an ethnically diverse population [33,34].

## Strengths and limitations

To the best of our knowledge, this study is the first to assess CC in GP trainees and GP trainers. In developing the questionnaire, we used a conceptual framework for CC that focuses on the patient-doctor interaction [15]. This framework has previously been used to develop a questionnaire to assess CC among youth health physician residents and their physician supervisors in the Netherlands [18] and among midwives in Europe [19]. We were therefore able to compare the results. We used a self-developed questionnaire that has not been validated. However, we used a diverse and broadly experienced team of experts to develop a questionnaire that resembles the practical, everyday issues related to the care of migrants in general practice. We also used the think-aloud method to test the content validity of the questionnaire. We believe that these aspects are the most important in achieving content validity, which is considered the most important characteristic of such an instrument [35,36].

Our questionnaire included medical cases to identify intended attitudes and culturally competent skills that the authors considered relevant to assessing CC in general practice beyond self-perception. However, the use of a questionnaire means that we were dependent on self-reported attitudes and culturally competent skills [18].

Self-reported questionnaires are prone to social desirability bias [37] and are weakly associated with observer-rated cultural competence [38–40]; therefore, the validity of self-reported cultural competence questionnaires has been questioned [41].

In order to measure CC skills more objectively, instruments are needed for the observation of CC in general practice, such as analysing videotaped consultations. A recent systematic review [41] reported that there are yet no suitable observation instruments available for objectively assessing all domains of CC in HCPs. In this review, five instruments were found in the literature, all of which had suboptimal psychometric properties and were mainly aimed at the assessment of attitude.

We cannot exclude selection bias; only a small number of trainers and trainees who answered our questionnaire indicated that they work in practices with a high proportion of migrants and ethnic minority patients. Approximately half of the respondents indicated having 10% or fewer migrant patients in their general practice. Nevertheless, we included a diverse study population with regard to, for example, the year of residency, the ethnicity of the respondents and their affinity for the care of migrant patients. In addition, the results of our questionnaire may not be generalisable to all general practices, as general practices and the GP training curriculum may differ between regions and countries.

## Conclusions and recommendations

This study identified gaps in cultural competence among GP trainees and GP trainers and highlighted the need for cultural competence training. Cultural competence appears to increase with experience and exposure to an ethnically diverse patient population. Practice-oriented education may therefore help to improve cultural competence. Further studies should explore how cultural competency in general practice can be improved through educational programmes within the GP training curricula in order to reduce ethnic disparities in health care. We also need validated instruments to test all aspects of cultural competence (i.e. knowledge, attitude and skills).

## Supplementary Material

Supplemental Material

Supplemental Material

Supplemental Material
