# Supplementary material for: Cultural competency of GP trainees and GP trainers: a cross-sectional survey study
Source: Scand J Prim Health Care. 2024 Feb 7;42(1):101–11. doi: 10.1080/02813432.2023.2293927 (PMC10851837; doi:10.1080/02813432.2023.2293927)
Supplement: Supplemental Material [file IPRI_A_2293927_SM5796.docx]

**Appendix 2.**

**Survey: *General practice care for people with a migrant background***

Dear GP trainee/ GP trainer,

We invite you to take part in this research survey, which examines cultural competences with regard to patient-oriented general practice healthcare for people with a migrant background.

The results of this questionnaire will be used to develop a “diversity” educational programme for the GP training curriculum. The questionnaire is anonymous, and the data cannot be traced back to individuals.

- You are free to stop completing the questionnaire at any moment. The data provided up to that point will still be kept and used for the research.
- No financial compensation is attached to the completion of this questionnaire.

*** I agree with the above points and consent to take part in this survey.**

- Yes
- No

**Design:**

The questionnaire comprises the following components:

Part 1: General questions

Part 2: Day-to-day practice

Part 3: Knowledge questions

Part 4: Training needs

Completing the questionnaire will take about 20 minutes.

**The questionnaire defines a number of terms as follows:**

- *Migrant*: in this questionnaire, migrants are held to be those people having a *non-western migrant background*. These are people originally from Turkey, Africa, Latin America and Asia. They include asylum seekers, refugees and the undocumented. Expatriates are not regarded as non-western migrants for the purposes of this questionnaire.
- *Functional illiteracy*: having so much difficulty with reading, writing and/or arithmetic that it is an impediment to daily life.
- *Health literacy*: the skills needed to obtain information on health, to understand this information, to assess it, and to use it when taking health-related decisions.

**Part 1: General questions**

1. What is your sex?

- Female
- Male
- Other / I’d rather leave this open

1. At which GP training institute are you affiliated as a GP trainee or GP trainer?

- Amsterdam UMC, location VUmc
- Amsterdam UMC, location AMC

1. What is your current general practice traineeship (only applicable for GP trainees)?

- Year 1 traineeship
- Year 3 traineeship

1. How many years of work experience had you gained as a (junior) doctor before you started the general practice programme?

……………………………………………………………………

1. How many years have you worked as a GP trainer (only applicable for GP trainers)?

………………………………………………………………………

1. How many years have you worked as a GP (only applicable for GP trainers?

………………………………………………………………………

1. In your estimation, what percentage of patients in the general practice where you are currently working as a GP trainee or GP have a migration background?

- ≤10%
- 10 – 25%
- 26 – 50%
- >50%

1. Looking at the general practice in which you are working, from which three countries did most of these non-western migrants originally come?

1.…………………………………………

2.…………………………………………

3.…………………………………………

1. Which of the following statements applies to you?

- My parents and I were all born in the Netherlands
- I was born in the Netherlands, but at least one of my parents and their family were born in another country, namely ………………………………………………………………………………
- My parents and I were born in another country, namely…………………………………

1. How much affinity would you say you have with the healthcare of migrants?

- Very strong affinity
- Strong affinity
- Neutral
- Little affinity
- No affinity

**To what extent would you agree with the following statements?**

Your answer is given on a scale of 1 to 5, where 1 = strongly disagree and 5 = strongly agree.

1. I have difficulty in providing healthcare for people with a non-western migrant background as a result of the language barrier and/or cultural differences.

- 1. Strongly disagree
- 2. Disagree
- 3. Neither agree nor disagree (neutral)
- 4. Agree
- 5. Strongly agree

1. To what extent do you feel equipped to provide good (culture-competent) healthcare to people with a non-western migrant background? Select the answer that that best applies to you (1= very poorly equipped and 10= extremely well equipped).

1 2 3 4 5 6 7 8 9 10

**Part 2: Day-to-day practice**

The following case studies and the accompanying multiple-choice questions reflect the kind of situations you could experience in your day-to-day practice.

**Your answers should reflect what you actually do in practice, not what you think would be best in theory. If you have never experienced the situation described, please indicate what you would probably think or do in such a situation.**

**Case 1:**

Mr el Mourabit (52) is of Moroccan origin. He has lived in the Netherlands for 35 years and speaks reasonably good Dutch (to a secondary vocational education level 1). He has had type 2 diabetes mellitus (T2DM) and hypertension for the last 2.5 years. Mr el Mourabit often arrives late or fails to show up for his check-ups with the POH (*praktijkondersteuner huisarts*, practice nurse). The POH informs you that it appears to be impossible to make Mr el Mourabit adhere to the metformin, gliclazide and NPH insulin medication he is now being prescribed. His HbA1c remains about 62 mmol/mol. The POH has a strong suspicion that Mr el Mourabit does not always take his medication regularly because, for instance, he often fails to pick it up on time from the pharmacy. The POH is also concerned about Mr el Mourabit’s obesity. He has been unable to lose weight; a number of past visits to a dietician have had no results. Mr el Mourabit is unwilling to be referred to a dietician.

1. **Which of the following scenarios come closest to your own thoughts? More than one answer may be given.**

- You are losing patience with this case as Mr el Mourabit is clearly refusing to follow the POH’s advice
- You propose that Mr el Mourabit be registered as a ‘care refuser’ because he is avoiding care
- Mr el Mourabit is non-adherent and needs once again to be given a clear, emphatic explanation of the importance of using medication in accordance with the doctor’s prescription
- *You wonder whether Mr el Mourabit’s poor glycaemic picture is related to limited health literacy, reducing his capacity for self-management********
- *You wonder whether Mr el Mourabit’s resistance to referral to a dietician might be related to inadequately culture-sensitive nutrition advice********
- *You wonder whether Mr el Mourabit’s disrupted sugar balance might be related to certain stressors he might be experiencing, such as debt or discrimination********

**Case 2:**

Ms Sardjoe is a 29-year-old woman of Hindustani Surinamese origin. She was born in the Netherlands, and has attained a secondary vocational education level 2. You experience no language barrier with her, but you suspect that she might be functionally illiterate.

1. **How would you tailor your communications to her suspected functional illiteracy? More than one answer may be given.**

- *I would use short sentences and simple words********
- I would ask mostly closed questions
- *I would avoid the use of metaphors********
- I would regularly ask: “Do you understand?”
- *I would have the patient repeat what I had just explained********
- I would limit the number of core issues/messages to 6 per consultation

**Case 3:**

Mr Yildiz (72) is of Turkish origin. He has been registered with your practice for five years. When he comes in he is usually accompanied by one of his children, who translate for him because his Dutch language skills are inadequate.

Mr Yildiz has presented with T2DM and mild decompensatio cordis. You recently referred Mr Yildiz to a lung specialist in connection with a persistent cough and unexplained weight loss. The pulmonologist has just called you to say that Mr Yildiz has been diagnosed with extensively metastasised lung cancer, with no therapeutic options. He also told you that Mr Yildiz has not yet been informed of this diagnosis and prognosis. Mr Yildiz’ children are completely opposed to the idea that their father be told of his misfortune.

13. **What does this case evoke in you? Indicate which of the following responses are most similar to your own. More than one answer may be given.**

- It annoys me that the family is deciding that the patient should not know about the diagnosis
- I consider it of the greatest importance that Mr Yildiz knows what to expect
- *I have sympathy for the family; they care about Mr Yildiz and are trying to protect him********
- *I want to know why the family does not want Mr Yildiz to know the truth********
- *I am curious what Mr Yildiz himself would like to know about the diagnosis and prognosis********
- I cannot agree to the family’s request; it is my duty as a doctor to inform the patient of his incurable lung cancer as honestly and fully as possible

**Follow-up case**

You have had a talk with Mr Yildiz’ children. It has become clearer to you that their request was prompted by their fears that that their father would lose all hope of recovery if he learned that he has an incurable lung cancer, and would soon die. The bad news might also worsen his health and make him depressed. Moreover, they believe that only Allah knows when a person will die, and that it is not the doctor’s place to voice an opinion on Mr Yildiz’ life expectancy.

1. **On the basis of this information, what would you now tell the family? More than one answer may be given.**

- You show understanding for their perspective, and explain that as a doctor you have a duty to tell their father honestly and fully about his illness and prognosis
- You explain that you cannot agree to their request, as only their father may decide whether he does or does not wish to be informed about his own diagnosis and prognosis
- You show understanding for their perspective, and agree to their request not to inform their father about his diagnosis and prognosis
- *You show understanding for their perspective, and explain that you wish to learn directly from their father whether he does or does not want to be informed about his diagnosis and prognosis********
- *You indicate that that you will not volunteer information about the prognosis, but you will not evade further questions about it from their father********
- *You show understanding for their perspective, and agree to the family’s wish to not talk in detail about life expectancy, but you will inform their father of his diagnosis********

After a long talk with the family it is agreed that you will tell Mr Yildiz about his diagnosis, but say nothing about his life expectancy. You plan a home visit, a few days later, to Mr and Mrs Yildiz. Their two daughters and a son, who speak good Dutch, have said they would like to be present.

1. **You wonder whether you ought to bring a professional interpreter to this ‘bad news’ interview. More than one answer may be given.**

- A professional interpreter is **not** needed, as Mr Yildiz’ children speak good Dutch
- A professional interpreter is **not** desirable in this situation, as professional interpreters have no bond of trust with the patient
- A professional interpreter is **not** desirable in such an emotionally charged situation, because in comparison to family members they can offer the patient no emotional support
- *A professional interpreter* ***is*** *needed in this situation, as interpreting the bad news might be emotionally stressful for the children********
- *A professional interpreter* ***is*** *needed in this situation, given the complexity of the issue********
- *A professional interpreter* ***is*** *needed in this situation, to increase the trustworthiness of the information given to the patient********

**As was already mentioned, you have agreed with Mr Yildiz’ family that you will tell him about the diagnosis, but not about the prognosis.**

1. **What will you tell Mr Yildiz during this ‘bad news’ interview? More than one answer may be given.**

- *You will tell him that he is terminally ill and that you cannot make him better********
- You will tell him that he has lung cancer and that you cannot help him
- You will tell him that he will die of this disease
- *You will ask him what he would like to know about the disease********
- *You will tell him that he is very ill and that you will do all you can to make sure he does not suffer********
- You will tell him that you have a duty to tell him of the fact that he has lung cancer

**Follow-up case**

You receive a panic-stricken telephone call from Mr Yildiz’ son: their father is not doing at all well. He has weakened considerably in recent days, and is hardly eating or drinking. You decide to make a house call. Mr Yildiz has started morphine medication and does not seem to be in pain. He is cachectic and is showing decreased responsiveness. Mr Yildiz is clearly in the terminal phase of his illness. The family is desperate. They cannot bear to see Mr Yildiz deteriorating in this way and refusing food and drink. As his daughter says: *“Nobody can stay alive without water. If we don’t do something now we will be letting him die.*” The family ask that Mr Yildiz be referred to hospital to receive an immediate infusion.

1. **What would you do if asked for this hospital referral? More than one answer may be given.**

- *I would explain to the family that Mr Yildiz is in the terminal phase of his illness, and that not eating or drinking is normal at that stage********
- I would explain to the family that Mr Yildiz is in the terminal phase of his illness, and that given his current situation he is not expected to live long
- *I would explain to the family that in Mr Yildiz’ situation an infusion would not help, and might even have adverse effects, such as shortness of breath********
- I would begin by stating that sending him to hospital or giving him an infusion would be medically useless and that I therefore cannot agree to their request
- *I would consider alternatives that might help to alleviate the family’s sense of guilt and helplessness, agreeing on a compromise if necessary********
- I would understand the family’s desperation and agree to send Mr Yildiz to hospital at his family’s request

**18.A. Have you ever used the services of a professional interpreter?**

- Yes
- No

1. **B. Select the option(s) you use in practice when working with a professional interpreter. If you have never worked with a professional interpreter, select the option(s) you would use. More than one answer may be given.**

- *I speak to the patient directly (“Where is the pain?”)********
- *I use short sentences********
- I give the interpreter space to take their own initiative during the interview
- I make effective use of the time taken for the interpreter to translate (e.g. by updating the patient dossier)
- *I avoid puns and quips********
- I ask the interpreter to elucidate any relevant cultural aspects

**Part 3: Knowledge questions**

The following questions concern your knowledge of healthcare for migrants. The questions are given in the form of a statement. For each statement you can select one of three possible answers: ‘correct’, ‘incorrect’, or ‘I don’t know’. **Please don’t make guesses!** We need to be able to identify any lacunae in the knowledge base to be able to develop an appropriate educational programme.

1. In the Netherlands, a large majority of functionally illiterate people have a migrant background.

- Correct
- *Incorrect********
- I don’t know

1. In the Netherlands, people with a low educational attainment level have a healthy life expectancy that is, on average, five years shorter than that of highly-educated people.

- Correct
- *Incorrect********
- I don’t know

1. Amongst people whose origins lie in sub-Saharan Africa, diuretics should **not** be prescribed as a preferred antihypertensive.

- Correct
- *Incorrect********
- I don’t know

1. Amongst people whose origins lie in sub-Saharan Africa, high blood pressure is best treated with an ACE inhibitor.

- Correct
- *Incorrect********
- I don’t know

1. When prescribing antidepressants and statins to people whose origins lie in Ethiopia, it is necessary to consider that they metabolise these drugs more slowly; the medication therefore needs to be given in lower doses.

- Correct
- *Incorrect********
- I don’t know

1. From a medical perspective, people with type 1 DM as well as those with type 2 DM cannot fast during Ramadan.

- Correct
- *Incorrect********
- I don’t know

1. In Islam, Muslims with a chronic illness are allowed to take oral medication during the fast.

- Correct
- *Incorrect********
- I don’t know

1. In patients with a migrant background, exploring MUPS using the SCEBS model (Somatic, Cognition, Emotion, Behaviour, Social) needs to be done in a different order than amongst patients of Dutch origin.

- *Correct********
- Incorrect
- I don’t know

1. Most of the cost of medically necessary GP care for undocumented (‘illegal’) patients can be compensated through the National Health Care Institute (CAK/Zorginstituut).

- *Correct********
- Incorrect
- I don’t know

**Ethnically specific diagnoses**

Certain ethnic groups are more likely to suffer from a range of medical conditions that seldom if ever appear in patients of Dutch origin. The following statements each comprise a summary description of the symptoms together with important information on the patient’s ethnic background. For each case, indicate which diagnosis you would rank higher in the DDx than for patients of Dutch origin. You can assume that all these patients have no other relevant medical history.

1. Mr Sardjoe (35), a Hindustani Surinamese man, presents with severe symptoms of fatigue and weakness.

- Differential diagnosis: ……………………………………………………………………………………………………………

1. Ms Adam (45) is a Muslim woman from Sudan who has been troubled by muscle pain and fatigue for a couple of years. The muscle pain is particularly evident in her upper legs, meaning that she has trouble using stairs and getting out of a chair.

- Differential diagnosis: ……………………………………………………………………………………………………………

1. Ms Kamara is originally from Sierra Leone in West Africa. She has lived in the Netherlands for a month, together with her five-month-old son. Ms Kamara is worried about her little boy: he has become less lively, is growing more slowly, and is pale.

- Differential diagnosis: ……………………………………………………………………………………………………………

1. Ms Arakelian, who is of Armenian origin, has come to the practice with her 11-year-old son, who has suffered from repeated attacks of stomach-ache and fever for the past six months. These attacks last from one to four days.

- Differential diagnosis: ……………………………………………………………………………………………………………

1. You are familiar with Ms Ali (34), a Somalian woman, as a patient with recurring urinary tract infections (UTI) and a chronic pain in her lower abdomen. She has come to the practice with, once again, dysuria and lower abdominal pain, with no fever. You ascertain an uncomplicated UTI, but what might be the underlying cause of recurring UTIs in Ms Ali?

- Differential diagnosis: ……………………………………………………………………………………………………………

1. Mr Özcan (30), of Turkish origin, has come to the practice several times over the past couple of years in connection with joint pain, and is now presenting with ulcers in his mouth and on his genitalia.

- Differential diagnosis: ……………………………………………………………………………………………………………

**Part 4: Training needs**

This final section of the survey is intended to identify the training you may already have received on a refresher day, and any needs you might have for future GP training with regard to patients with a migrant background.

**34. For each of the following subjects, indicate on a scale of 1 to 3 the extent to which these topics were covered during a refresher day or during continuing education.** Give your answers on a scale of 1 to 3, where: 1 = not covered; 2 = inadequately covered; 3 = adequately covered.

**35. In the last column, tick the boxes for those topics about which you would like to receive more education.**

1 2 3

**I need education on:**

**Not covered Inadequately covered Adequately covered**

a. Ethnic differences in pharmacotherapy 0 0 0 0

b. Specific or more frequent comorbidities

in migrant populations 0 0 0 0

c. Culture-specific subjects such as diabetes and 0 0 0 0

Ramadan; culture-sensitive lifestyle advice

d. The treatment and supervision of migrants 0 0 0 0

with mental health problems, such as MUPS

e. Palliative care for people with a migrant background 0 0 0 0

f. Specific aspects of care for refugees and care 0 0 0 0

organisations for undocumented people

g. Communication skills to overcome language barriers and 0 0 0 0

functional illiteracy, such as clarifying the request for help,

exploring concerns and emotions, and giving

explanations tailored to the patient’s level of literacy

h. Communication skills for holding consultations 0 0 0 0

with an interpreter (e.g. maintaining control)

i. Developing an ethnically/culturally sensitive attitude when 0 0 0 0

providing appropriate care to migrants

j. Dealing with ethical dilemmas specific to healthcare 0 0 0 0
 for migrants

K. Tools that can be used when providing healthcare to 0 0 0 0

migrants and/or functionally illiterate people

L. Are there any other subjects relevant to diversity training that you would like to mention here?

--------------------------------------------------------------------------------------------------------------------

1. Could you indicate the extent to which you feel equipped to give good, culture-competent healthcare to people with a migrant background? Select the answer that most applies to you (1 = very poorly equipped, 10 = very well equipped).

1 2 3 4 5 6 7 8 9 10

Would you be willing to provide input into the design of the education programme we plan to develop based on the results of this survey?

- Yes, and I can be reached by email at ……………………………………………………
- No

**Note: Your email address will immediately be separated from your answers to this survey in order to safeguard the anonymity of your responses.**

**Are there any remarks you would like to make that arise from this questionnaire?**

------------------------------------------------------------------------------------------------------------------------------------------------------------------

------------------------------------------------------------------------------------------------------------------------------------------------------------------

------------------------------------------------------------------------------------------------------------------------------------------------------------------

------------------------------------------------------------------------------------------------------------------------------------------------------------------

**Thank you very much for your valuable contribution to this research survey!**

****=Correct or appropriate answer.***
