# Supplementary material for: Cultural competency of GP trainees and GP trainers: a cross-sectional survey study
Source: Scand J Prim Health Care. 2024 Feb 7;42(1):101–11. doi: 10.1080/02813432.2023.2293927 (PMC10851837; doi:10.1080/02813432.2023.2293927)
Supplement: Supplemental Material [file IPRI_A_2293927_SM5788.docx]

**Appendix 1. Description of the adapted Seeleman et al. questionnaire**

|  | Knowledge | Attitudes | Skills |
| --- | --- | --- | --- |
| Original domains of cultural competence defined in the framework of Seeleman et al. | 1. Knowledge of differential effects of treatment in various ethnic groups.  2. Knowledge of epidemiology and manifestation of diseases in various ethnic groups. | 3. Awareness of how culture shapes individual behaviour and thinking.  4. Awareness of the social contexts in which specific ethnic groups live.  5. Awareness of one’s own prejudices and tendency to stereotype.^a^ | 6. Ability to transfer information in a way the patient can understand and to know when to seek external help with communication.  7. Ability to adapt to new situations flexibly and creatively. |
| What we measured  Domain | a) General knowledge of ethnic minority care provision.  b) Knowledge of ethnic specific diagnosis. | Culturally competent consultation attitudes | Culturally competent skills |
| Operationalisation for the questionnaire:  What we measured in the questionnaire (the numbers between brackets refer  to the domains of cultural competencies defined in the framework) | - General knowledge of ethnic minority care provision (1).  - Knowledge of prevalent disease among people with a migration background such as diabetes, (2). | - Awareness of how culture shapes individual behaviour and thinking and curiosity about understanding or learning more about patients’ perspectives (3).  - Awareness of the role of social context and patients’ specific factors such as health literacy in relation to for example non-adherence (4). | - The ability to adapt communication to patients’ [health] literacy, to assess when external help with communication is needed [e.g. interpreter]; and how to work with an interpreter during consultation (6).  - The ability to compromise with patients or their family as needed and to adapt flexibly and creatively to new situations such as an ethical dilemmas during palliative care (7). |
| Description of questions and response options | - Nine statements on knowledge of ethnic minority care provision (true/ false/ I do not know).*  - Six short cases on knowledge of ethnic specific diagnosis (open questions: i.e. a differential diagnosis). ** | Three multiple choice questions in which respondents were asked what they would do or think in real life, based on medical cases/ scenario’s which are relevant to General Practice (each question has six options from which respondents are asked to choose one or more options in accordance to the way they would react or think in real life). *** | Five multiple choice questions on what respondents would do in real life, based on medical cases/ scenario’s which are relevant to General Practice (each question has six options from which respondents are asked to choose one or more options in accordance to the way they would react in real life).**** |

^a^This specific subsection of attitudes is not operationalised in our questionnaire.

*Items on general knowledge were scored per correct answer [‘correct’=1 point, ‘incorrect’=0, and ‘I do not know’=0]. Range score 0-9.

**Items regarding ethnic specific diagnosis were scored for the correct differential diagnosis (‘correct’=1 point, ‘incorrect’=0). Two questions of these items had two correct answers. In those cases the differential diagnosis were scored based on the most important diagnosis (+0.67 points) and the second correct diagnosis (+0.33 points). Participants were given a total of one point if both correct differential diagnosis were provided. Range score 0-6.

***0.33 points per correct (appropriate) option, with a maximum score of 1 point per item (range score 0-3).

****0.33 points per correct (appropriate) option, with a maximum score of 1 point per item (range score 0-5).
