# Supplementary material for: Cultural competency of GP trainees and GP trainers: a cross-sectional survey study
Source: Scand J Prim Health Care. 2024 Feb 7;42(1):101–11. doi: 10.1080/02813432.2023.2293927 (PMC10851837; doi:10.1080/02813432.2023.2293927)
Supplement: Supplemental Material [file IPRI_A_2293927_SM1152.docx]

**Appendix 3. Amount of covered education training per training institute according to GP trainees and trainers.**

|  |  | N (%) | N (%) | N (%) | N (%) |
| --- | --- | --- | --- | --- | --- |
| Topics on care of migrants |  | GP trainees  VUmc (N=46) | GP trainees  AMC (N=46) | GP trainers  VUmc  (N=92) | GP trainers  AMC  (N=94) |
| Prevalent diseases among migrants | - adequately covered - inadequately covered - not covered | 0 (0%)  21 (45.7%)  25 (54.3%) | 0 (0%)  30 (65.2%)  15 (32.6%) | 11 (12.0%)  59 (64.1%)  19 (20.7%) | 9 (9.6%)  57 (60.6%)  23 (24.5%) |
| Ethnical differences in pharmacotherapy | - adequately covered - inadequately covered - not covered | 0 (0%)  12 (26.1%)  34 (73.9%) | 1 (2.2%)  22 (47.8%)  22 (47.8%) | 5 (5.4%)  44 (47.8%)  41 (44.6%) | 3 (3.2%)  47 (50.0%)  37 (39.4%) |
| Cultural specific topics (such as diabetes and Ramadan) | - adequately covered - inadequately covered - not covered | 4 (8.7%)  20 (43.5%)  22 (47.8%) | 15 (32.6%)  12 (26.1%)  18 (39.1%) | 27 (29.3%)  52 (56.5%)  11 (12.0%) | 21 (22.3%)  57 (60.6%)  9 (9.6%) |
| Intercultural palliative care | - adequately covered - inadequately covered - not covered | 8 (17.4%)  14 (30.4%)  24 (52.2%) | 12 (26.1%)  20 (43.5%)  13 (28.3%) | 17 (18.5%)  49 (53.3%)  24 (26.1%) | 9 (9.6%)  50 (53.2%)  27 (28.7%) |
| Care for refugees and undocumented migrants | - adequately covered - inadequately covered - not covered | 1 (2.2%)  6 (13.0%)  39 (84.8%) | 2 (4.3%)  11 (23.9%)  32 (69.6%) | 5 (5.4%)  36 (39.1%)  48 (52.2%) | 5 5.3%)  34 (36.2%)  51 (54.3%) |
| Communication skills 1* | - adequately covered - inadequately covered - not covered | 6 (13.0%)  24 (52.2%)  16 (34.8%) | 8 (17.4%)  23 (50.0%)  14 (30.4%) | 23 (25.0%)  47 (51.1%)  20 (21.7%) | 20 (21.3%)  50 (53.2%)  20 (21.3%) |
| How to cope with ethical dilemmas | - adequately covered - inadequately covered - not covered | 5 (10.9%)  19 (41.3%)  21 (45.7%) | 7 (15.2%)  20 (43.5%)  18 (39.1%) | 10 (10.9%)  49 (53.3%)  30 (32.6%) | 7 (7.4%)  50 (53.2%)  32 (34.0%) |
| Developing a culturally sensitive attitude | - adequately covered - inadequately covered - not covered | 1 (2.2%)  17 (37.0%)  27 (58.7%) | 2 (4.3%)  23 (50.0%)  20 (43.5%) | 8 (8.7%)  44 (47.8%)  37 (40.2%) | 13 (13.8%)  42 (44.7%)  33 (35.1%) |
| Psychological conditions (such as MUPS)** | - adequately covered - inadequately covered - not covered | 4 (8.7%)  20 (43.5%)  22 (47.8%) | 8 (17.4%)  24 (52.2%)  13 (28.3%) | 10 (10.9%)  47 (51.1%)  32 (34.8%) | 12 (12.8%)  43 (45.7%)  33 (35.1%) |
| Information on specific tools | - adequately covered - inadequately covered - not covered | 4 (8.7%)  19 (41.3%)  23 (50.0%) | 7 (15.2%)  20 (43.5%)  17 (37.0%) | 12 (13.0%)  45 (48.9%)  32 (34.8%) | 16 (17.0%)  37 (39.4%)  36 (38.3%) |
| Communication skills 2*** | - adequately covered - inadequately covered - not covered | 0 (0%)  5 (10.9%)  41 (89.1%) | 5 (10.9%)  9 (19.6%)  30 (65.2%) | 11 (12.0%)  25 (27.2%)  55 (59.8%) | 9 (9.6%)  31 (33.0%)  51 (54.3%) |

*Communication skills to overcome language barriers and functional illiteracy, such as clarifying the request for help, exploring concerns and emotions, and giving explanations tailored to the patient’s level of literacy.

**Medically unexplained physical symptoms (MUPS).

***Communication skills for holding consultations with an interpreter (e.g. maintaining control).
